# Characterization of epidermal growth factor receptor (*EGFR*) P848L, an unusual *EGFR* variant present in lung cancer patients, in a murine Ba/F3 model

**DOI:** 10.1002/2211-5463.12702

**Published:** 2019-09-07

**Authors:** Bhaswati Sarcar, Nicholas T. Gimbrone, Gabriela Wright, Lily L. Remsing Rix, Edna R. Gordian, Uwe Rix, Alberto A. Chiappori, Gary W. Reuther, Pedro G. Santiago‐Cardona, Teresita Muñoz‐Antonia, William Douglas Cress

**Affiliations:** ^1^ Department of Molecular Oncology H. Lee Moffitt Cancer Center and Research Institute Tampa FL USA; ^2^ Department of Drug Discovery H. Lee Moffitt Cancer Center and Research Institute Tampa FL USA; ^3^ Department of Tumor Biology H. Lee Moffitt Cancer Center and Research Institute Tampa FL USA; ^4^ Department of Thoracic Oncology H. Lee Moffitt Cancer Center and Research Institute Tampa FL USA; ^5^ Biochemistry and Cancer Biology Divisions Ponce Health Sciences University Puerto Rico

**Keywords:** Ba/F3 cells, *EGFR* mutation, Janus kinase inhibitor, lung cancer, Tyr 1045, tyrosine kinase inhibitor

## Abstract

Lung cancer patients with mutations in epidermal growth factor receptor (EGFR) benefit from treatments targeting tyrosine kinase inhibitors (TKIs). However, both intrinsic and acquired resistance of tumors to TKIs are common, and EGFR variants have been identified that are resistant to multiple TKIs. In the present study, we characterized selected EGFR variants previously observed in lung cancer patients and expressed in a murine bone marrow pro‐B Ba/F3 cell model. Among these EGFR variants, we report that an exon 20 deletion/insertion mutation S768insVGH is resistant to erlotinib (a first‐generation TKI), but sensitive to osimertinib (a third‐generation TKI). We also characterized a rare exon 21 germline variant, EGFR P848L, which transformed Ba/F3 cells and conferred resistance to multiple EGFR‐targeting TKIs. Our analysis revealed that P848L (a) does not bind erlotinib; (b) is turned over less rapidly than L858R (a common tumor‐derived EGFR mutation); (c) is not autophosphorylated at Tyr 1045 [the major docking site for Cbl proto‐oncogene (c‐Cbl) binding]; and (d) does not bind c‐Cbl. Using viability assays including 300 clinically relevant targeted compounds, we observed that Ba/F3 cells transduced with EGFR P848L, S768insVGH, or L858R have very different drug‐sensitivity profiles. In particular, EGFR P848L, but not L858R or S768insVGH, was sensitive to multiple Janus kinase 1/2 inhibitors. In contrast, cells driven by L858R, but not by P848L, were sensitive to multikinase MAPK/extracellular‐signal‐regulated kinase (ERK) kinase and ERK inhibitors including EGFR‐specific TKIs. These observations suggest that continued investigation of rare TKI‐resistant EGFR variants is warranted to identify optimal treatments for cancer.

**Abbreviations**
Chx: cycloheximide
del: deletion
EGFR: epidermal growth factor receptor
ERK: extracellular‐signal‐regulated kinase
IL‐3: interleukin‐3
ins: insertion
NSCLC: non‐small‐cell lung cancer
TKI: tyrosine kinase inhibitor
WT: wild‐type

Cancer‐associated kinase domain variants in the epidermal growth factor receptor (*EGFR*) are common in non‐small‐cell lung cancer (NSCLC) as they can result in the increased ligand‐independent kinase activity of the receptor. The most common and well‐characterized activating *EGFR* mutations are various in‐frame deletion (del) around the LREA region of amino acids 747–750 within exon 19 (del19) and exon 21 mutations resulting in the L858R substitution 1, 2, 3. Another 10% of well‐characterized *EGFR* mutations involve exons 18 and 20 1, 3. The mutational status of *EGFR* and its downstream signaling molecules have implications for treatment response. First‐generation tyrosine kinase inhibitors (TKIs), including gefitinib and erlotinib, significantly improve progression‐free survival in stage IV lung cancer patients who are positive for activating *EGFR* mutations such as L858R and del19 4. However, the effectiveness of individual TKIs may depend upon the exact nature of the *EGFR* mutations 5. Subgroup analyses have suggested that the benefit of *EGFR* TKIs is greater in *EGFR* mutants with deleted exon 19 6, 7, compared to the exon 21 L858R substitution, although these findings are not universal 8, 9. Unlike *EGFR* exon 19 del and L858R mutants, most NSCLCs with *EGFR* exon 20 insertion (ins) mutations do not respond to gefitinib or erlotinib and the mechanism of drug resistance remains unresolved 2.

Although most *EGFR*‐positive NSCLC patients show initial benefit upon TKI treatment, acquired resistance ultimately leads to disease progression within ~ 1 year. Among the several molecular mechanisms of resistance to *EGFR* TKIs, the ‘gatekeeper’ mutation T790M is best characterized and observed in 60% of NSCLC patients 10. To specifically target T790M and other resistant mutations, second‐ and third‐generation TKIs have been developed, such as Gilotrif (afatinib), AZD 9291 (osimertinib), and CO‐1686 (rociletinib) 11, 12, 13. Not surprisingly, novel mutations are emerging, and among these, the exon 19 L747P and exon 20 C797S mutations are found to impute resistance to gefitinib and osimertinib, respectively 13, 14, 15. While we can predict the drug sensitivity of the majority of cancer‐associated *EGFR* variants, there are less common *EGFR* variants 16, 17, 18, 19, 20, 21, 22, 23, 24 that remain poorly characterized, and thus, their clinical relevance remains unclear. In the present study, we have used a Ba/F3 cell line model to rapidly assess the transforming activity and drug sensitivity of a selected cohort of *EGFR* variants, and included some that are reported previously 16, 17, 18, 19, 20, 21, 22, 23, 25 and some novel variants from a recently published cohort of Hispanic lung cancer patients 24. Results show that most of the selected *EGFR* variants transform Ba/F3 cells and a few were found to be resistant to TKIs. The most interesting example is an exon 21 germline variant (P848L) that is resistant to all EGFR‐targeted TKIs tested, but is sensitive to multiple Janus kinase inhibitors (JAKis). Taken together, our findings suggest that more work is needed to evaluate the nature of rare *EGFR* variants in lung cancer and that JAKis may represent a new strategy to inhibit rare variants of *EGFR* that do not respond to *EGFR*‐targeted TKIs.

## Methods

### Cell culture, transient transfection, retrovirus production, retroviral infection, and generation of stable lines

Gene Oracle Inc. (Santa Clara, CA, USA) used a semi‐synthetic site‐specific mutagenesis approach and constructs we provided to generate *EGFR* wild‐type (WT) and mutant constructs in a pBabe‐puro retroviral vector. Each mutant construct was sequence‐verified. 293T cells were grown in Dulbecco's modified Eagle's medium (Gibco, Gaithersburg, MD, USA) with 10% FBS (Gibco), 100 units·mL^−1^ penicillin and streptomycin, and 0.1 mmol·L^−1^ MEM nonessential amino acids (Mediatech, Inc., Holly Hill, FL, USA). Ba/F3 cells were grown in RPMI containing 10% FBS, penicillin/streptomycin, and 5% WEHI‐3 cell‐conditioned medium as a source of interleukin‐3 (IL‐3; Corning, Corning, NY, USA). 293T cells were transfected with empty pBabe‐puro vector, WT *EGFR*, or mutant *EGFR* pBabe‐puro constructs, along with viral packaging vectors (pVPack; Agilent Technologies, Wilmington, DE, USA), using Lipofectamine 2000 following the manufacturer's instructions (Invitrogen, Carlsbad, CA, USA). Retrovirus was generated, and retroviral infections were performed as previously described 26. Briefly, stable polyclonal cell lines were generated by selecting pBabe‐puro virus‐infected cells with puromycin (1.0 μg·mL^−1^). IL‐3 was reduced by half every 2 days over a period of 2 weeks. EGFR protein expression levels in IL‐3‐independent cells were evaluated by western blot analysis. *EGFR* variant expression in Ba/F3 cells was also confirmed by target‐specific *EGFR* sequencing. Briefly, RNA was extracted from mutant‐expressing Ba/F3 cell lines per standard protocol (RNeasy Mini Kit; Qiagen, Germantown, MD, USA). Total RNA was reverse‐transcribed into single‐stranded cDNA using iScript^TM^ cDNA Synthesis Kit (Bio‐Rad, Hercules, CA, USA). The cDNA regions of EGFR (exons 18–21) were then PCR‐amplified and sequenced with EGFR‐specific forward (5′‐AGATCAAAGTGCTGGGCTCC‐3′) and reverse (5′‐TCTTTCTCCGCACCCAG‐3′) primers. During sequence verification, it was observed that only V834L‐expressed cell line has generated additional mutation at the position E804 to K, which might be due to the effect of multiple subculturing. Growth curves were generated by plating Ba/F3 cells at 50 000 cells·mL^−1^, and viable cells were counted by trypan blue exclusion every 2 days until cell density reached 106 cells·mL^−1^ at which point the cells were replated at 50 000 cells·mL^−1^. Pooled stable cells that were transformed to IL‐3 independence were used for drug‐sensitivity experiments.

### Drugs and antibodies

Erlotinib, osimertinib, and AZD1480 were obtained from Selleckchem (Houston, TX, USA), CL‐387 785 was purchased from Enzo Life Sciences (Farmingdale, NY, USA), and ruxolitinib was purchased from ChemieTek (Indianapolis, IN, USA). One and ten millimolar stock solutions of TKIs were prepared in DMSO. The following antibodies from Cell Signaling (Danvers, MA, USA) were used: EGFR phospho‐Tyr 992, phospho‐Tyr1045, phospho‐Tyr1068, phospho‐Tyr1173, EGFR (D38B1XP), phospho‐p44/42 MAPK (Erk1/2) (Thr202/Tyr204) (20G11), p44/42 MAPK (Erk1/2), phospho‐STAT3 (Tyr 705), STAT3, phospho‐STAT5 (Tyr694), JAK1, JAK2, JAK2‐pY‐1007/1008, and c‐Cbl GRB2. STAT5 (c‐17) was purchased from Santa Cruz Biotechnology (Dallas, TX, USA), and anti‐β‐actin was from Sigma (St Louis, MO, USA).

### Cell proliferation and viability assays

Ba/F3 cells stably expressing mutant EGFR proteins were seeded in 12‐well plates (100 000 cells·well^−1^), incubated either with vehicle control (DMSO) or with the indicated concentrations of inhibitors for 3 days. Generally, cell viability was determined using a hemocytometer and the trypan blue dye exclusion assay. The sensitivity of *EGFR* variants to TKIs was evaluated using the high‐throughput CellTiter‐Glo assay. Briefly, 1000 cells·well^−1^ were seeded into black clear‐bottom 384‐well plates. Vehicle control (final DMSO concentration of 0.4%) and either erlotinib or osimertinib were added immediately after seeding. Cells were incubated in a 37 °C 5% CO_2_ incubator for 3 days prior to analysis with CellTiter‐Glo luminescent reagent (Promega, Madison, WI, USA). Data were recorded using M5 Spectramax plate reader (Molecular Devices, San Jose, CA, USA), cell viability was normalized to vehicle‐treated wells, and data were fit to a sigmoidal dose–response curve using graphpad prism 6 (San Diego, CA, USA). All the experiments were performed in triplicate. A further drug screening study was extended using 300 clinically relevant small molecule drugs and probes of various classes including, but not limited to EGFRi, JAKi, mTORi, MAPK/extracellular‐signal‐regulated kinase (ERK) kinase inhibitors (MEKi) PI3Ki, AKTi, and ERKi. Mutant‐expressing Ba/F3 cells were treated for 72 h with 0.1 and 1 μm of each compound in biological duplicate, and viability was determined using CellTiter‐Glo as described previously with minor modifications 27.

### Western blotting

Whole‐cell protein extraction was performed by pelleting the cells by centrifugation in cold 1X PBS and lysed in NP40 buffer [50‐mmol·L^−1^ Tris (pH 8.0), 150‐mmol·L^−1^ NaCl, 1.0% NP40, and 1X Proteinase Inhibitor Cocktail Set (Calbiochem, San Diego, CA, USA) and phosphatase inhibitor (Santa Cruz Biotechnology)]. Protein concentration was determined using the Pierce BCA protein assay reagent (Thermo Scientific, Waltham, MA, USA). Protein lysates (40 μg) were resolved by SDS/PAGE and transferred to Immobilon‐P membranes (Millipore, Danvers, VA, USA), and incubated overnight in corresponding primary antibody at 4 °C. Secondary antibodies conjugated to horseradish peroxidase (GE Healthcare, Waukesha, WI, USA) were used, and chemiluminescence (Thermo Fisher Scientific) was used for detection.

### Immunoprecipitation

Ba/F3 cells were serum‐starved for 24 h prior to treatment with 100‐ng·mL^−1^ EGF (Roche Diagnostics) for 30 min. Cells were harvested and washed once with PBS, and lysates were prepared in IP lysis buffer (0.025‐m Tris, 0.15‐m NaCl, 0.001‐m EDTA, 1% NP40, 5% glycerol; pH 7.4) from Pierce Biotechnology (Rockford, IL, USA) with Complete Mini Protease Inhibitor Cocktail (Roche Diagnostics, Basel, Switzerland). Immunoprecipitation was carried out using Pierce Immunoprecipitation Kit (Pierce). Briefly, the EGFR, or c‐Cbl, antibody was allowed to bind the protein A/G plus agarose in the resin column. One milligram of lysate was precleared with control agarose resin and then subjected to immunoprecipitation on a rotation shaker at 4 °C overnight. Antigens bound to the antibody‐coupled agarose beads were washed with IP lysis buffer and with 1X conditioning buffer provided in the kit. Proteins were then eluted with sample buffer and subjected to SDS/PAGE, and western blotting was carried out with either anti‐c‐Cbl or anti‐EGFR antibodies (Cell Signaling).

### Cycloheximide treatment

Ba/F3 cells expressing *EGFR* variants were treated either with DMSO (the carrier) or with 1‐μm erlotinib for the indicated times, and lysates were collected for western blotting. Alternatively, cells were treated either with DMSO or erlotinib (1 μm) first, and 16 h later, protein synthesis was blocked with freshly prepared cycloheximide (50‐μg·mL^−1^ CHX; Sigma). Western blotting was carried out for total EGFR and actin to visualize protein expression.

### Fluorescence microscopy

Ba/F3 cells (0.5 × 10^6^) were treated with DMSO or 1 μm erlotinib for 18 h. From the treated plates, 1 × 10^5^ cells were suspended in PBS and collected on cytospin slides (Lab Scientific, Inc., Highlands, NJ, USA) by centrifugation (3 min, 500 r.p.m.). Cells were fixed with 100% prechilled methanol, permeabilized with 0.2% Triton X‐100, blocked with 10% goat serum (Invitrogen) in PBS, and incubated overnight at 4 °C with the anti‐EGFR antibody (Cell Signaling). Cells were incubated with secondary antibody (Alexa Fluor 594 anti‐rabbit IgG; Invitrogen) and were counterstained and mounted with antifade containing 4′,6‐diamidino‐2‐phenylindole (DAPI; Invitrogen). Micrograph images were acquired in the Moffitt Analytical Microscopy Core with a Leica SP8 AOBS laser scanning confocal microscope through a 63X/1.4NA Plan Apochromat oil immersion objective lens (Leica Microsystems CMS GmbH; Wetzlar, Germany). 405, 488, and 552 laser lines were applied to excite the samples, and tunable emissions were used to minimize cross talk between fluorochromes. Images for each sample were captured with photomultiplier detectors and las af software version 2.6 (Leica Microsystems).

### Erlotinib pull‐down assay

Ba/F3 L858R and Ba/F3 P848L cells were lysed as described previously 28. Immobilization of c‐erlotinib and affinity purifications were performed essentially as described previously 29. Experiments were performed in duplicate using 5 mg protein per pull‐down for both the cell lysates. For competition controls, total cell lysates (TLC) were pretreated with 20 μm erlotinib for 30 minutes before addition of drug beads. The SDS/PAGE analysis was performed as described previously 29.

### Phospho‐RTK array

A mouse phospho‐receptor tyrosine kinase (p‐RTK) array kit including 39 RTKs was purchased from R&D systems (Minneapolis, MN, USA). Serum‐starved and EGF (100 ng·mL^−1^)‐induced cells were cultured in 10‐cm^2^ culture plates. Cells were lysed, and 300 μg of protein was used for western blotting according to the manufacturer's protocol. Activated receptors were matched according to the phospho‐RTK array coordinates.

## Results

### Expression and tyrosine phosphorylation status of selected EGFR variants

**Figure 1:**
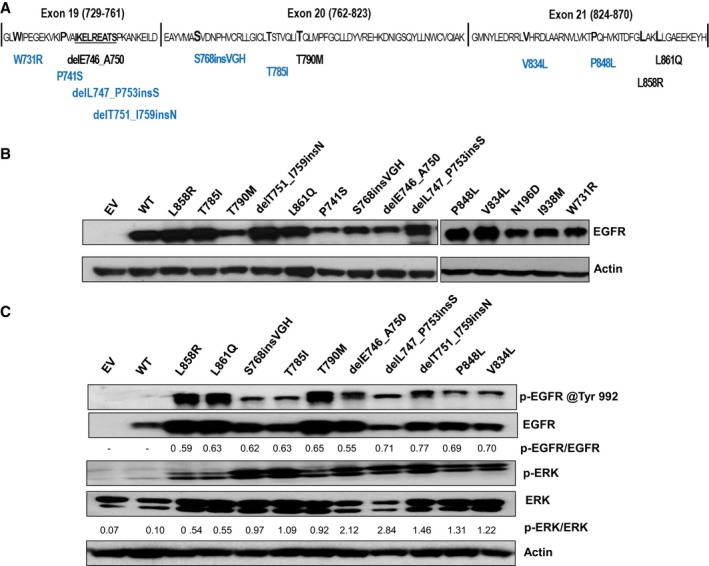
Expression and tyrosine phosphorylation status of selected EGFR variants. (A) This schematic represents tumor‐derived *EGFR* variants characterized within exons 19–21 of the *EGFR* tyrosine kinase domain. Common well‐characterized mutations are indicated in black below the central amino acid sequence, and rare/novel *EGFR* variants are indicated below the central amino acid sequences in blue. Two variants (I938M and N196D) included in this study (see Table 1) reside outside the kinase domain. (B) Western blotting demonstrates the expression levels of selected *EGFR* proteins in Ba/F3 cells. Actin served as a loading control. (C) Western blots for phospho‐EGFR and phospho‐ERK were used to measure activated EGFR (via phospho‐EGFR Tyr992 antibody, p‐EGFR) and activated ERK (via phospho‐ERK Thr202/Tyr204 antibody, p‐ERK). Total EGFR protein, ERK, and actin served as loading controls. Relative band intensities were determined with ImageJ software, and phospho‐EGFR and phospho‐ERK were normalized against total EGFR and total ERK (indicated ratios are in arbitrary units). Results are the representative of three independent experiments.

Table 1 and Fig. 1A highlight the selected *EGFR* variants that were cloned into retroviral vectors and expressed in the murine pro‐B cell line, Ba/F3. In the absence of an exogenous activated form of *EGFR*, Ba/F3 cells are dependent on IL‐3 for growth 25. Figure 1B reveals the expression levels of the various *EGFR* variant proteins following retroviral transduction, but prior to IL‐3 withdrawal. Upon IL‐3 withdrawal, Ba/F3 cells infected with either pBabe‐puro empty vector or pBabe‐*EGFR* WT failed to grow, as expected 25. Likewise, four tumor‐derived *EGFR* variants W731R, P741S, N196D, and I938M also failed to drive the IL‐3‐independent growth of Ba/F3 cells. These variants that were not autophosphorylated did not lead to downstream ERK activation (data not shown), indicating that they are likely carrier mutations. The remaining *EGFR* variants transformed Ba/F3 cells and demonstrated similar doubling time to that of the parental cells provided IL‐3 (Table 1), indicating that these cancer‐derived variants are indeed *EGFR* activating mutations. Figure 1C demonstrates that *EGFR* Tyr992, one of the several known sites of autophosphorylation within the cytoplasmic C‐terminal tail of EGFR 30, was not phosphorylated in WT *EGFR*, as expected, whereas activating mutants were all phosphorylated (with some variation). A downstream EGFR mediator, ERK, was also shown to be phosphorylated/activated at Thr202/Tyr204 in cells expressing the oncogenic mutants, but not WT *EGFR* (Fig. 1C).

**Table 1:** Characterization of *EGFR* variants. ND, not determined

*EGFR* variant	Exon	Ba/F3 transforming	Doubling time (h)	Erlotinib, IC50, nm	Osimertinib, IC50, nm	CL‐387 785, IC50, nm	References
delL747_P753insS	19	Yes	20.73	1.5	ND	ND	21
delT751_I759insN	19	Yes	19.6	9.3	ND	ND	21
delE746_A750	19	Yes	20.62	4.3	ND	ND	1, 2, 3, 4, 6, 24
L861Q	21	Yes	18.95	36	ND	ND	5, 24, 31
T785I	20	Yes	18.75	12	ND	ND	16, 17, 18, 24, 42
V834L	21	Yes	17.29	99	ND	ND	24, 43, 44
L858R	21	Yes	20.29	16	2.6	0.20	1, 2, 3, 4, 24
T790M	20	Yes	19.89	> 1000	13	250	10, 11, 13, 59, 60
S768insVGH	20	Yes	18.85	814	74	63	24
P848L	21	Yes	18.00	> 1000	> 715	> 1000	22, 32, 33
W731R	19	No					20, 24
P741S	19	No					19, 24
N196D^a^	2–4	No					24
I938M^a^	23	No					24

a N196D and I938M lie outside the *EGFR* kinase domain. N196D was observed in a carcinoid tumor, and I938M was observed in a squamous, both were from cohort of Hispanic lung cancer patients 24.

### Sensitivity of EGFR mutants to TKIs

The sensitivity of the transforming *EGFR* variants to erlotinib, osimertinib, and CL‐387 785 (three clinically relevant drugs targeting *EGFR*) was explored next. As representative experiments show in Fig. S1A and Table 1, the common activating mutants, L858R and delE746_A750, and the less common del mutants, delL747_P753insS and delT751_I759insN, are very sensitive to erlotinib with IC_50_ values of < 20 nm. In contrast, the exon 21 mutation L861Q (IC_50_, 36 nm) is only moderately sensitive to erlotinib, as reported earlier 5, 31. Among the rare mutants examined, T785I (IC_50,_ 12 nm) is sensitive to erlotinib and V834L (IC_50_, 99 nm) is somewhat resistant. S768insVGH (IC_50_, 814 nm) and P848L (IC_50_, > 1000 nm) were highly resistant to erlotinib. We treated these two erlotinib‐resistant mutants with osimertinib and CL‐387 785 along with L858R and T790M as controls (Fig. S1B,C). As expected, L858R is sensitive to both drugs, whereas T790M is resistant to CL‐387 785 (IC_50_, 250 nm), but sensitive to osimertinib (IC_50_, 13 nm). Rare mutant S768insVGH was sensitive to osimertinib (IC_50,_ 74 nm) and CL‐387 785 (IC_50_, 63 nm; Table 1 and Fig. S1D). Interestingly, P848L was resistant to osimertinib (IC_50_, 715 nm) and to CL‐387 785 (IC_50_, > 1000 nm; Table 1 and Fig. S1E).

### Biochemical characterization of P848L

**Figure 2:**
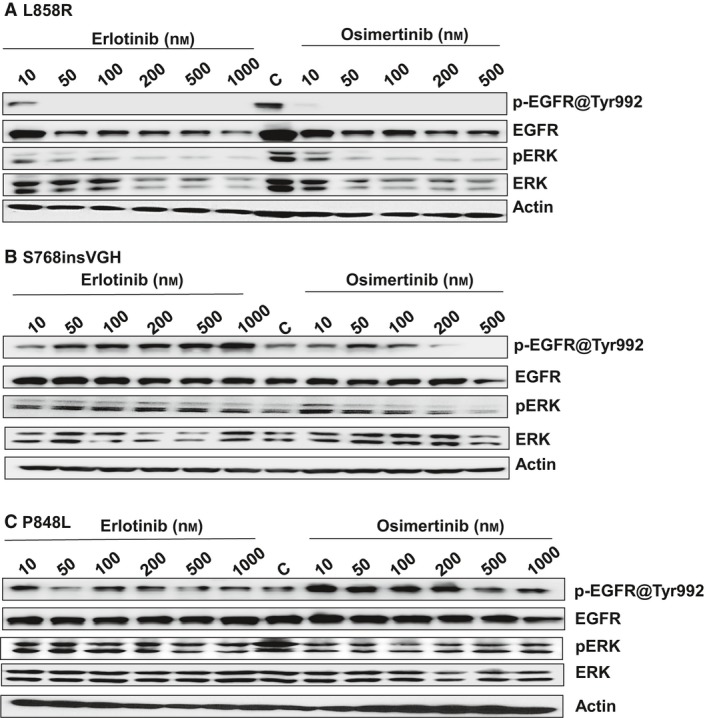
Sensitivity of *EGFR* variants to TKIs. Three derived cell lines, (A) L858R, (B) S768insVGH, and (C) P848L, were subjected to western blots to measure activated EGFR (via phospho‐EGFR Tyr992, p‐EGFR) and activated ERK (via phospho‐ERK Thr202/Tyr204, p‐ERK) after treating for 24 h with the indicated concentrations of erlotinib and osimertinib (nm). Total EGFR protein, ERK, and actin served as loading controls in all three independent experiments.

P848L has been reported earlier as a novel germline *EGFR* variant, and cancer patients do not respond to the *EGFR* TKIs 23, 32, 33, 34. To explore the mechanism of P848L's resistance to the tested TKIs, and to better understand how the biological effects of P848L differ from other activating mutations, L858R‐, S768insVGH‐, and P848L‐expressing cells were treated with increasing doses of erlotinib and osimertinib. Autophosphorylation of EGFR at Tyr 992 and ERK signaling was measured by western blotting. P848L is autophosphorylated even at 1000‐nm erlotinib or osimertinib (Fig. 2C), whereas Tyr 992 phosphorylation of L858R (which is sensitive to both drugs) is completely blocked at 50 nm of either drug (Fig. 2A). Neither erlotinib nor osimertinib was able to completely inhibit the activation of ERK signaling in P848L mutant, but were able to block ERK signaling in L858R at 50 nm. Autophosphorylation of S768insVGH (Fig. 2B) and autophosphorylation of the downstream ERK were resistant to erlotinib, but sensitive to osimertinib (inhibition observed at 200 nm). Erlotinib treatment reduced L858R expression in a dose‐dependent manner, but did not change the expression of mutants S768insVGH or P848L. Rapid downregulation of L858R protein in lung cancer cell lines following erlotinib treatment is in accordance with the previous findings 35.

**Figure 3:**
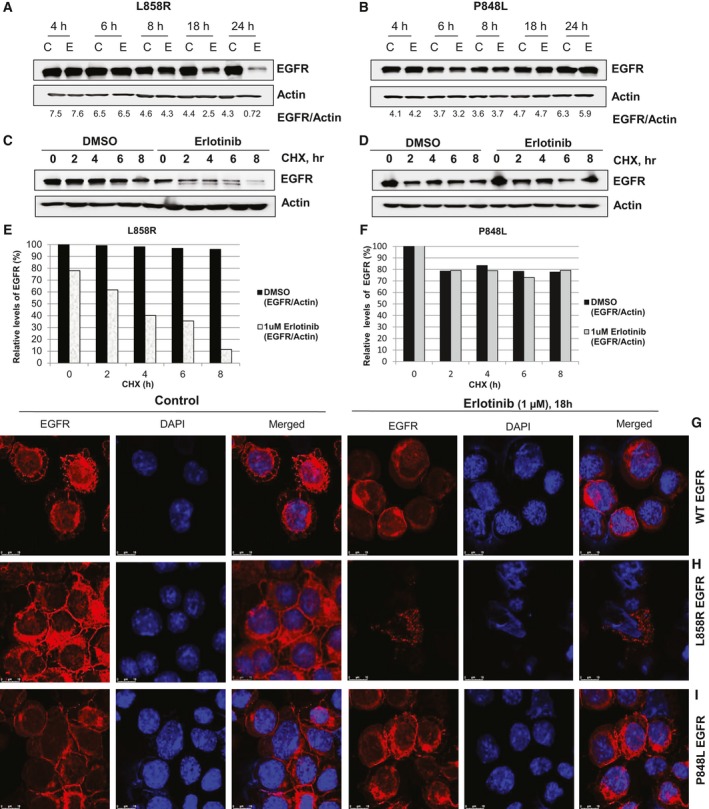
Erlotinib treatment reduces the expression of the L858R protein, with no effect on P848L expression. (A) Ba/F3 L858R and (B) Ba/F3 P848L cells were treated with either DMSO (C—control) or 1 μm erlotinib (E) and were subjected to western blotting over time, as indicated. The blots were scanned, and the EGFR bands were normalized against actin; relative values were quantified using ImageJ software, and indicated ratios are in arbitrary units. (C) L858R and (D) P848L cells were treated, as indicated, with erlotinib for 16 h followed by 50 μg·mL^−1^ CHX to stop protein synthesis, and levels of indicated proteins were determined by western blot analysis followed by a semi‐quantitative analysis by scanning of blots. Graphical plots of L858R (E) and P848L (F) are representative of two separate experiments, and relative level of EGFR band intensities was measured using ImageJ software and normalized against actin. (G) Ba/F3 WT EGFR, (H) Ba/F3 L858R, and (I) Ba/F3 P848L cells were treated, as indicated, for 18 h and subjected to confocal microscopy. Cells were fixed and stained with EGFR antibody (red) and DAPI (blue, DNA dye). Representative 63X images (Scale bar = 10 μm) are presented (see Methods for details), and the experiment was done twice.

To further characterize the effect of erlotinib treatment on protein stability, cells expressing P848L or L858R, as a control, were treated with either DMSO or 1‐μm erlotinib and cells were harvested at 4, 6, 8, 18, and 24 h post‐treatment and subjected to western blotting (Fig. 3A–D). Approximately, more than 80% of L858R EGFR was lost by 24 h, whereas P848L protein levels remained almost unaffected. Next, L858R and P848L Ba/F3 cells were treated with DMSO or 1 μm erlotinib for 16 h followed by treatment with 50 μg·mL^−1^ CHX to stop protein synthesis. As shown in Fig. 3A,C erlotinib treatment led to a reduction of L858R expression in a time‐dependent manner, but failed to reduce the expression of P848L protein (Fig. 3B,D). Quantification graph using imagej software (Madison, WI, USA) is showing the remaining level of EGFR in L858R (Fig. 3E) and the level of EGFR in P848L (Fig. 3F) cells treated with erlotinib.

Nuclear localized EGFR has been associated with disease progression and worse overall survival in numerous cancers 36. Because of this, Ba/F3 cells expressing EGFR WT (grown with IL‐3), L858R, or P848L were also subjected to anti‐EGFR immunofluorescence after treatment with either DMSO or 1 μm erlotinib for 18 h (Fig. 3G–I). In untreated cells, WT, L858R, and P848L were primarily localized to the outer plasma membrane. As expected from the western blotting, we observe a significant decrease in immunofluorescence of L858R following erlotinib treatment (Fig. 3H). Conversely, P848L immunofluorescence was undiminished following erlotinib treatment (Fig. 3I). Nuclear translocation of EGFR after erlotinib treatment was not observed in either L858R‐ or P848L‐expressing Ba/F3 cells (Fig. 3H–I). As expected, WT EGFR was not affected by erlotinib (Fig. 3G).

**Figure 4:**
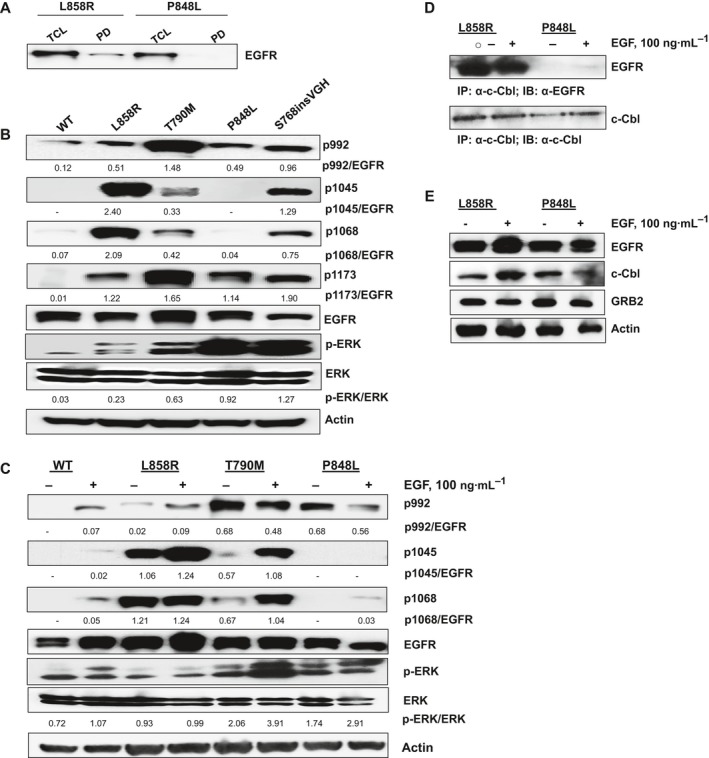
Biochemical characterization of P848L. (A) Lysates (5 mg) from L858R and P848L transduced Ba/F3 cells were subjected to erlotinib‐Sepharose pull‐down assay and 1/3 of the erlotinib‐bound protein subjected to western blotting, see ‘PD’ lanes [61]. Ten micrograms of TCL were loaded for reference. (B) Ba/F3 cells expressing EGFR WT, L858R, T790M, S768insVGH, and P848L were subjected to western blot analysis to probe for phosphorylated EGFR at Tyr 992, Tyr 1045, Tyr 1068, and Tyr 1173 residues. (C) Ba/F3 cells expressing WT and mutant EGFRs are serum‐starved for 24 h and then stimulated with 100 ng·mL^−1^ of EGF for 30 min. Pellets were harvested, and lysates were prepared and subjected to western blotting using the indicated antibodies. Total EGFR and actin served as loading controls for all the experiments. Indicated band intensities of phosphorylated EGFRs and phosphorylated ERKs in Fig. (B) and (C) were normalized against total EGFR and total ERK, respectively, using ImageJ software (indicated ratios are in arbitrary units). (D) L858R‐ and P848L‐expressing Ba/F3 cells were serum‐starved for 24 h prior to stimulation with 100 ng·mL^−1^ of EGF. Control cells (−) were serum‐starved, but not stimulated with EGF. One milligram of whole‐cell lysates were immunoprecipitated with a c‐Cbl antibody, followed by western blotting anti‐EGFR (upper panel) or anti‐c‐Cbl antibody which recognizes c‐Cbl, as control (lower panel). (E) As described in (A), L858R‐ and P848L‐expressing Ba/F3 cells were serum‐starved for 24 h prior to stimulation with EGF (100 ng·mL^−1^). Fifty micrograms of cell lysate was subjected to western blotting using the indicated antibodies to EGFR, c‐Cbl, and GRB2. Actin serves as loading control. The data are representative of three independent experiments.

Next, using a previously described resin‐linkable erlotinib analog 37 we performed an affinity pull‐down assay. Lysates (5 mg) from L858R‐ and P848L‐transduced Ba/F3 cells were subjected to erlotinib‐Sepharose pull‐down assay and 1/3 of the erlotinib‐bound protein subjected to western blotting. Ten micrograms of the TCL was loaded for reference. The data (Fig. 4A) reveal that erlotinib pulled down detectable amounts of L858R, as expected, but could not pull down detectable P848L. This observation suggests that the P848L mutation causes drug resistance by reducing the affinity to erlotinib. Autophosphorylation plays an important role in EGFR signaling; therefore, we examined tyrosine phosphorylation of L858R, T790M, P848L, and S768insVGH at residues Y992, Y1045, Y1068, and Y1173 and their respective downstream signaling ERK. The basal level of phosphorylation varied in different mutants. In contrast to T790M, tyrosine phosphorylation of L858R at Y1045 and Y1068 was higher compared to Y992 and Y1173. Phosphorylation levels at different tyrosine residues of S768insVGH were in general indistinguishable (Fig. 4B). In contrast, P848L showed a consistent and similar level of autophosphorylation at Y992 and Y1173, like L858R (Fig. 4B), but was not autophosphorylated at Y1045 and to a lesser extent at Y1068. ERK is also differentially phosphorylated in these mutants (Fig. 4B). We next sought to determine whether the receptor autophosphorylations of the various mutants are ligand‐dependent. For this experiment, cells were serum‐starved for 24 h and then treated with EGF (100 ng·mL^−1^) for 30 min. As shown in Fig. 4C, WT EGFR autophosphorylation is activated at all three tyrosine residues upon EGF treatment, as expected. EGF treatment also activated T790M autophosphorylation at Y1045 and Y1068, but did not affect Y992, which was constitutively phosphorylated without ligand stimulation. EGF treatment induced low, but detectable, P848L phosphorylation at Y1068, but was unable to induce detectable Y1045 phosphorylation. EGF treatment also induced the L858R phosphorylation at Y992, Y1045, and Y1068 residues (Fig. 4C), and downstream ERK signaling was also elevated when induced with ligand either in WT EGFR or in mutant EGFRs, as expected (Fig. 4C).

Y1045 is the docking site for the Cbl E3 ubiquitin ligase, and phosphorylation at Y1045 of EGFR is necessary for Cbl binding, and for receptor downregulation and ubiquitination *in vitro* 30, 38. Cbl is recruited through interactions with the GRB2 adaptor protein. Therefore, lack of phosphorylation of Y1045 indicates less affinity for Cbl binding. To further explore whether the differences in the phosphorylation status of tyrosine residues in the C‐terminal tail of P848L affect Cbl binding, co‐immunoprecipitation was performed either with an anti‐c‐Cbl antibody or with anti‐EGFR antibody and subsequent western blotting with anti‐EGFR or anti‐c‐Cbl. As shown in Fig. 4D and Fig. S2A, c‐Cbl was physically associated with L858R both in the absence or in the presence of EGF as reported earlier 39. In contrast, constitutively activated P848L does not physically associate with c‐Cbl under the same experimental conditions. Expression of total c‐Cbl, GRB2, and EGFR was confirmed by western blot (Fig. 4E) in both the cell lines. Therefore, our findings suggest that the unphosphorylated form of Tyr 1045 in the P848L mutant contributes to the stability of this mutant by reducing its binding to Cbl.

Next, to evaluate the potential activation of bypass pathways in P848L‐driven Ba/F3 cells, we examined the phosphorylation level of other RTKs, using a mouse phospho‐RTK array kit, in either serum‐starved or EGF‐induced L858R‐ and P848L‐expressing Ba/F3 cells. Phospho‐RTK array experiments revealed a similar level of phosphorylated EGFR in both EGF‐induced cell lines and less phosphorylated PDGFR in P848L (Fig. S2C) compared to L858R (Fig. S2B)‐expressing Ba/F3 cells, but there was no significant activation of other tested tyrosine receptor family kinases in L858R or in P848L mutants.

### EGFR variants differ in sensitivity to targeted compounds

To identify optimal therapeutic approaches for the identified EGFR TKI‐resistant variants L858R and S768insVGH, we performed viability assays in the presence of 0.1 and 1 μm of nearly 300 clinically relevant targeted compounds. Most of the compounds in the library are FDA‐approved or are in clinical development and have multiple target classes such as EGFRi, JAKi, mTORi, MEKi PI3Ki, AKTi, and ERKi. Unsupervised hierarchical clustering revealed that the three tested mutants showed distinct biological responses toward the 300 tested compounds at each dose (Fig. S3A,B). To narrow the focus, the first principal component was calculated for both the 0.1 μm (Fig. S3C) and 1 μm (Fig. S3D) treatments and the top 30 drugs contributing to the variance were compared. Again, this analysis suggested that all three variants had distinct sensitivities; however, Fig. S3D reveals that JAK inhibitors were highly selective for inhibition of P848L.

**Figure 5:**
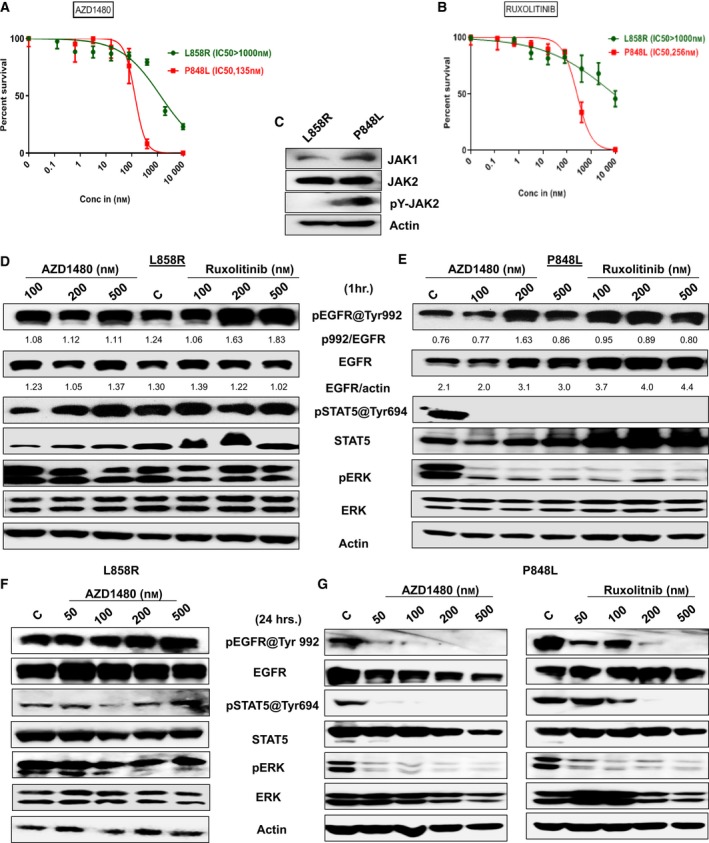
*EGFR* variants differ in sensitivity to targeted compounds. (A, B) The sensitivity of *EGFR* variants (L858R and P848L) to JAK1/2Is (AZD1480 and ruxolitinib) was evaluated using a high‐throughput CellTiter‐Glo. Errors bars represent standard deviation. The specific drug concentrations used were 10 000, 2000, 400, 80, 16, 3.2, 0.64, and 0.128 nm. Briefly, 1000 cells·well^−1^ were seeded into black clear‐bottom 384‐well plates. Inhibitors were added immediately after seeding. Cells were incubated for 3 days prior to analysis with CellTiter‐Glo luminescent reagent. Plates were read on an M5 Spectramax plate reader; cell viability was normalized to vehicle‐treated wells and fit to a sigmoidal dose–response curve using graphpad prism 6. Experiments were performed in triplicate. (C) Total expressions of JAK1, JAK2, and phospho JAK2 (pY‐JAK2) in Ba/F3‐expressing L858R and P848L were measured by western blotting, and actin serves as loading control. Ba/F3 cells expressing either L858R or P848L *EGFR* mutation were treated for 1 h. (D, E) and 24 h. (F, G) with the indicated concentrations of JAKi's AZD1480 and ruxolitinib, and autophosphorylations of EGFR at Tyr992, p‐STAT5 at Tyr 694, and p‐ERK were measured by western blotting. Total EGFR, STAT5, ERK, and actin served as loading controls for each experiment. Experiments were done in triplicate, and quantification of phosphorylated EGFR against total EGFR was done using ImageJ software, and using the same software, total EGFR was normalized against actin. Indicated ratios are in arbitrary units. Experiments were performed in triplicate.

In Fig. 5A,B, we specifically explore two clinically relevant JAK1/2 inhibitors AZD1480 and ruxolitinib in viability assays. As expected from the screening results, L858R is resistant to both the drugs (IC_50,_ > 1000 nm) likely via direct phosphorylation of STAT5, as reported 40; however, P848L is sensitive; AZD1480 IC_50_ is 135 nm, and ruxolitinib IC_50_ is 256 nm. Next, we sought to determine whether JAK2 is activated in P848L but not in L858R. Data in Fig. 5C demonstrate that P848L, and not L858R, induces a high level of phosphorylated JAK2 (Fig. 5C). The basal level of JAK1 and JAK2 expressions was also equivalent in both L858R and P848L cell lines (Fig. 5C). Next, we explored the biological significance of JAK1/2 inhibition on P848L mutants and assessed the status of downstream signaling molecules, such as ERK and STAT3/5 30 in Ba/F3 P848L and L858R cells. We treated both cell lines with AZD1480 and ruxolitinib (Fig. 5D–G), as well as with EGFR TKI erlotinib (Fig. S4A,B). JAK1/2 inhibition blocked STAT3/5 and ERK signaling downstream of P848L EGFR (Fig. 5E and Fig. S4C) within 1 h in a dose‐dependent manner. Interestingly, within 1 h of treatment with either drug the phosphorylated EGFR and total EGFR have increased in P848L‐expressing cells, but with no significant change observed in L858R mutants, which is in accordance with the previous findings of increased EGFR signaling by JAK2 inhibition in established TKI‐resistant NSCLC cells *in vitro* and in primary tumors due to the surface abundance of EGFR 41. Phosphorylation of P848L EGFR and phosphorylation of downstream signaling molecules signal transduction and activator of transcription (STAT) and ERK are inhibited within 24 h (Fig. 5G) with increasing doses of either drug. As expected, neither AZD1480‐ nor ruxolitinib‐inhibited L858R autophosphorylation (Fig. 5D,F) inhibition of downstream effector signaling pathways ERK and JAK/STAT was minimal at both time intervals tested (Fig. 5F and Fig. S5A). At the same time, autophosphorylation of P848L (Fig. S5B) and autophosphorylation of the downstream effectors STAT3/5 and ERK seemed to decrease insignificantly but completely inhibited in L858R EGFR (Fig. S5A)‐expressing Ba/F3 cells when treated with increasing doses of erlotinib within 1 h of treatment.

## Discussion

The current work addresses the molecular characterization of selected *EGFR* variants listed in Table 1 using the Ba/F3 transformation system. While we recognize the limitations of this approach, over 1000 publications have utilized Ba/F3 cells as a very powerful *in vitro* model system to study RTKs, such as EGFR. Since most novel EGFR mutations are identified from nucleic acids derived from fixed tissues, it is generally necessary to create novel artificial expression vectors corresponding to these sequences that can be expressed in cells at relatively consistent levels in a high‐throughput manner. Ba/F3 cells provide a way to systematically measure the transforming capacity of newly identified kinase mutations, and then to profile drug candidates and compound libraries in high‐throughput fashion. Since the Ba/F3 cells are completely dependent upon the activity of the expressed oncoprotein for growth, off‐target drug effects are completely accounted for by comparing with cells grown on IL‐3. The vigorous growth and the relatively high levels of protein expression of oncogenes in the Ba/F3 system also facilitate mechanistic studies, when they are called for. Four of the rare *EGFR* variants characterized (P741S, W731R, N196D, and I938M) are likely passenger mutations as those mutants failed to transform Ba/F3 cells. Two of these, P741S and W731R, have been previously reported, but not functionally characterized. W731R was reported earlier in a Japanese adenocarcinoma patient where it co‐occurred with an established activating point mutation, G719C 20. The P741S mutation (in exon 19) was reported previously as a single mutation in basal cell and breast carcinoma 19. In our previous study 24, the P741S variant co‐occurred with T785I variant in the same patient. Since T785I variant transformed Ba/F3 cells while the P741S variant did not, T785I is likely the driver mutation when these two variants coexist. The mutations N196D and I938M, both of which lie outside the core EGFR kinase domain, may be passenger mutations as well, since N196D and I938M did not transform Ba/F3 cells.

Exon 20 mutations are often associated with resistance to TKIs 10. Among the exon 20 mutations characterized, T785I was sensitive to erlotinib (IC_50_, 12 nm), whereas S768insVGH was insensitive (IC_50_, 814 nm). The T785I mutation has been previously observed in various cancers 16, 17, 18, 42, but none of these studies addressed whether it was a driving mutation or the sensitivity of the patients to TKIs. Interestingly, a novel del/ins mutation in exon 20, S768insVGH, was sensitive to osimertinib (IC_50_, 74 nm), but fairly insensitive to first‐generation TKIs, which supports previous findings that exon 20 del/ins mutations are resistant to the first‐generation TKIs 1. The exon 21 mutation V834L has been previously observed 24, 43, 44, but has not been characterized in detail. In Ba/F3 cells, V834L was transforming and was moderately sensitive to erlotinib (IC_50_, 99 nm), suggesting that patients with this variant are cancer‐causing and that the patient might benefit from erlotinib or a higher order TKI.

In our most unexpected result, a previously identified 22 *EGFR* variant, P848L, was found to be transforming and resistant to multiple *EGFR* TKIs. P848L was first reported as a novel germline variant in a lung adenocarcinoma patient who progressed on gefitinib 22. An early study examined P848L *in vitro* and observed that it was not autophosphorylated at Y‐1092 and did not activate AKT 45 concluding that P848L is likely a polymorphism and not an activating mutation. A later *in vitro* study 33 found P848L to be autophosphorylated and resistant to gefitinib, relative to L858R. Taken together, these results suggest that the biological activity of P848L may depend on the cellular context.

The population frequency of the P848L variant is very low; only 5 individuals among 121 292 tested have the variant1
http://exac.broadinstitute.org/variant/7-55249071-C-T, search performed July 24, 2019. (for reference the T790M gatekeeper variant, which is also transforming in the Ba/F3 system, is observed in 56 healthy individuals among 120 958 tested). Among cancer patients, P848L has been described in 16 carcinoma patients in Catalogue of Somatic Mutations in Cancer, and according to the available data, the frequency of P848L mutation found in cancer patients is about 0.6%.2
https://cancer.sanger.ac.uk/cosmic/mutation/overview?xml:id=22943, search performed July 24, 2019. There is an interesting clinical case study, which reported that *EGFR* P848L is a passenger SNP in *cis* position with the much more common EGFR L858R 46.

In clinical studies, P848L has been reported in patients that showed no response to *EGFR* TKIs 23, 32, 33, 34. One study demonstrates that it co‐occurred as a somatic mutation with L858R in a primary lung adenocarcinoma and its lymph node metastasis 47. A primary cell line generated from a NSCLC patient tissues with a P848L mutation formed tumors in nude mice and showed anchorage‐independent growth in soft agar 48. Although these clinical cases do not prove a causative role for P848L, our current work suggests that P848L could be an oncogenic contributor 49 and responsible for the poor response observed in these patients to first‐generation TKIs and thus worthy of biological characterization.

The very C‐terminal portion of the *EGFR* receptor, following the kinase domain, is a long tail segment with more than 200 residues that contains several tyrosine residues that undergo autophosphorylation and serve as docking sites for effector proteins that transmit signals further downstream 50. We demonstrate that the P848L is not phosphorylated at Y1045. The lack of Y0145 phosphorylation appears to abolish P848L's capacity to bind c‐Cbl, which normally leads to receptor ubiquitination and degradation following EGFR activation 38. This is not unprecedented as one study 39 showed that EGFRvIII, an EGFR variant bearing an internal in‐frame del in its external domain 51, is unphosphorylated or hypophosphorylated at Tyr 1045 and fails to associate with c‐Cbl, with concomitant failure to undergo ubiquitination and degradation.

Viability assay using a library of drugs revealed that P848L‐driven Ba/F3 cells are specifically sensitive to JAK1/2 inhibitors and resistant to TKIs, unlike the acquired T790M mutation which shows sensitivity to osimertinib 11. JAK/STAT signaling pathway has been implicated by several groups as a modulator of response and resistance to EGFR TKIs 41, 52, 53, 54. JAK2 inhibition abrogates proliferation in NSCLC cell lines, including those that are TKI‐resistant 55, and several reports showed that inhibition of STAT3 and STAT5 suppresses the growth of cancer cells and enhances the sensitivity to anticancer drugs in multiple types of cancers 30, 56, 57, 58. We find that JAK1/2 inhibitors are able to specifically attenuate EGFR receptor signaling in P848L‐expressing Ba/F3 cells and also inhibit two parallel downstream signaling pathways MAPK/ERK and JAK/STAT. This finding implies that TKI‐resistant P848L *EGFR* variant can be targeted using a JAK/STAT pathway inhibitors.

Our results support the conclusions that the rare *EGFR* germline variant P848L contributes to cellular transformation and predicts a lack of response to clinically relevant TKIs and that patients with the P848L variant may benefit from JAK inhibitors.

## Conflict of interest

The authors have no conflict of interest to disclose.

## Author contributions

Conception and design: BS, NTG, WDC, TM‐A, PGS‐C, and AAC; Development of methodology: BS, GB, and GWR; Acquisition of data: BS, LLRR, GW, and ERG; Writing, review, and/or revision of the manuscript: BS, NTG, WDC, GW, LLRR, GWR, PGS‐C, and TMA; Study supervision: WDC.

## Supporting information


**Fig. S1.** Sensitivity status of *EGFR* mutants to first and third generation TKIs.
**Fig. S2.** P848L does not physically associate with c‐Cbl.
**Fig. S3.** L858R, S768insVGH, and P848L differ in sensitivity to targeted compounds.
**Fig. S4.** Inhibition of STAT3 signaling upon treatment with JAK1/2 inhibitors in L858R and P848L expressing Ba/F3 cells.
**Fig. S5.** Kinase activity and downstream signaling of L858R and P848L upon treatment with erlotinib for 1 h.

## References

[1] Sharma SV , Bell DW , Settleman J and Haber DA (2007) Epidermal growth factor receptor mutations in lung cancer. Nat Rev Cancer 7, 169–181. 17318210 10.1038/nrc2088

[2] Yasuda H , Kobayashi S and Costa DB (2012) EGFR exon 20 insertion mutations in non‐small‐cell lung cancer: preclinical data and clinical implications. Lancet Oncol 13, e23–e31. 21764376 10.1016/S1470-2045(11)70129-2

[3] Yasuda H , Park E , Yun CH , Sng NJ , Lucena‐Araujo AR , Yeo WL , Huberman MS , Cohen DW , Nakayama S , Ishioka K *et al* (2013) Structural, biochemical, and clinical characterization of epidermal growth factor receptor (EGFR) exon 20 insertion mutations in lung cancer. Sci Transl Med 5, 216ra177. 10.1126/scitranslmed.3007205 PMC3954775 24353160

[4] Watanabe S , Minegishi Y , Yoshizawa H , Maemondo M , Inoue A , Sugawara S , Isobe H , Harada M , Ishii Y , Gemma A *et al* (2014) Effectiveness of gefitinib against non‐small‐cell lung cancer with the uncommon EGFR mutations G719X and L861Q. J Thorac Oncol 9, 189–194. 24419415 10.1097/JTO.0000000000000048 PMC4132025

[5] Kobayashi Y and Mitsudomi T (2016) Not all epidermal growth factor receptor mutations in lung cancer are created equal: Perspectives for individualized treatment strategy. Cancer Sci 107, 1179–1186. 27323238 10.1111/cas.12996 PMC5021039

[6] Zhang Y , Sheng J , Kang S , Fang W , Yan Y , Hu Z , Hong S , Wu X , Qin T , Liang W *et al* (2014) Patients with exon 19 deletion were associated with longer progression‐free survival compared to those with L858R mutation after first‐line EGFR‐TKIs for advanced non‐small cell lung cancer: a meta‐analysis. PLoS ONE 9, e107161. 25222496 10.1371/journal.pone.0107161 PMC4164616

[7] Yang JC , Wu YL , Schuler M , Sebastian M , Popat S , Yamamoto N , Zhou C , Hu CP , O'Byrne K , Feng J *et al* (2015) Afatinib versus cisplatin‐based chemotherapy for EGFR mutation‐positive lung adenocarcinoma (LUX‐Lung 3 and LUX‐Lung 6): analysis of overall survival data from two randomised, phase 3 trials. Lancet Oncol 16, 141–151. 25589191 10.1016/S1470-2045(14)71173-8

[8] Rosell R , Carcereny E , Gervais R , Vergnenegre A , Massuti B , Felip E , Palmero R , Garcia‐Gomez R , Pallares C , Sanchez JM *et al* (2012) Erlotinib versus standard chemotherapy as first‐line treatment for European patients with advanced EGFR mutation‐positive non‐small‐cell lung cancer (EURTAC): a multicentre, open‐label, randomised phase 3 trial. Lancet Oncol 13, 239–246. 22285168 10.1016/S1470-2045(11)70393-X

[9] Wu YL , Zhou C , Hu CP , Feng J , Lu S , Huang Y , Li W , Hou M , Shi JH , Lee KY *et al* (2014) Afatinib versus cisplatin plus gemcitabine for first‐line treatment of Asian patients with advanced non‐small‐cell lung cancer harbouring EGFR mutations (LUX‐Lung 6): an open‐label, randomised phase 3 trial. Lancet Oncol 15, 213–222. 24439929 10.1016/S1470-2045(13)70604-1

[10] Yu HA , Arcila ME , Rekhtman N , Sima CS , Zakowski MF , Pao W , Kris MG , Miller VA , Ladanyi M and Riely GJ (2013) Analysis of tumor specimens at the time of acquired resistance to EGFR‐TKI therapy in 155 patients with EGFR‐mutant lung cancers. Clin Cancer Res 19, 2240–2247. 23470965 10.1158/1078-0432.CCR-12-2246 PMC3630270

[11] Cross DA , Ashton SE , Ghiorghiu S , Eberlein C , Nebhan CA , Spitzler PJ , Orme JP , Finlay MR , Ward RA , Mellor MJ *et al* (2014) AZD9291, an irreversible EGFR TKI, overcomes T790M‐mediated resistance to EGFR inhibitors in lung cancer. Cancer Discov 4, 1046–1061. 24893891 10.1158/2159-8290.CD-14-0337 PMC4315625

[12] Janne PA , Yang JC , Kim DW , Planchard D , Ohe Y , Ramalingam SS , Ahn MJ , Kim SW , Su WC , Horn L *et al* (2015) AZD9291 in EGFR inhibitor‐resistant non‐small‐cell lung cancer. N Engl J Med 372, 1689–1699. 25923549 10.1056/NEJMoa1411817

[13] Wang S , Cang S and Liu D (2016) Third‐generation inhibitors targeting EGFR T790M mutation in advanced non‐small cell lung cancer. J Hematol Oncol 9, 34. 27071706 10.1186/s13045-016-0268-z PMC4830020

[14] Thress KS , Paweletz CP , Felip E , Cho BC , Stetson D , Dougherty B , Lai Z , Markovets A , Vivancos A , Kuang Y *et al* (2015) Acquired EGFR C797S mutation mediates resistance to AZD9291 in non‐small cell lung cancer harboring EGFR T790M. Nat Med 21, 560–562. 25939061 10.1038/nm.3854 PMC4771182

[15] Huang J , Wang Y , Zhai Y and Wang J (2018) Non‐small cell lung cancer harboring a rare EGFR L747P mutation showing intrinsic resistance to both gefitinib and osimertinib (AZD9291): a case report. Thorac Cancer 9, 745–749. 29673089 10.1111/1759-7714.12637 PMC5983145

[16] Wheler JJ , Falchook GS , Tsimberidou AM , Hong DS , Naing A , Piha‐Paul SA , Chen SS , Fu S , Stephen B , Fok JY *et al* (2013) Aberrations in the epidermal growth factor receptor gene in 958 patients with diverse advanced tumors: implications for therapy. Ann Oncol 24, 838–842. 23139256 10.1093/annonc/mds524 PMC4110484

[17] de Biase D , Visani M , Malapelle U , Simonato F , Cesari V , Bellevicine C , Pession A , Troncone G , Fassina A and Tallini G (2013) Next‐generation sequencing of lung cancer EGFR exons 18‐21 allows effective molecular diagnosis of small routine samples (cytology and biopsy). PLoS ONE 8, e83607. 24376723 10.1371/journal.pone.0083607 PMC3871524

[18] Chang YL , Wu CT , Shih JY and Lee YC (2011) Unique p53 and epidermal growth factor receptor gene mutation status in 46 pulmonary lymphoepithelioma‐like carcinomas. Cancer Sci 102, 282–287. 21070477 10.1111/j.1349-7006.2010.01768.x

[19] Ye Q , Qi F , Bian L , Zhang SH , Wang T and Jiang ZF (2017) Circulating‐free DNA mutation associated with response of targeted therapy in human epidermal growth factor receptor 2‐positive metastatic breast cancer. Chin Med J (Engl) 130, 522–529. 28229982 10.4103/0366-6999.200542 PMC5339924

[20] Koyama N , Jinn Y , Takabe K , Yoshizawa M , Usui Y , Inase N , Miyake S , Yoshizawa Y , Hagiwara K and Kanazawa M (2006) The characterization of gefitinib sensitivity and adverse events in patients with non‐small cell lung cancer. Anticancer Res 26, 4519–4525. 17201173

[21] Lee VH , Tin VP , Choy TS , Lam KO , Choi CW , Chung LP , Tsang JW , Ho PP , Leung DK , Ma ES *et al* (2013) Association of exon 19 and 21 EGFR mutation patterns with treatment outcome after first‐line tyrosine kinase inhibitor in metastatic non‐small‐cell lung cancer. J Thorac Oncol 8, 1148–1155. 23945384 10.1097/JTO.0b013e31829f684a

[22] Sequist LV , Joshi VA , Janne PA , Bell DW , Fidias P , Lindeman NI , Louis DN , Lee JC , Mark EJ , Longtine J *et al* (2006) Epidermal growth factor receptor mutation testing in the care of lung cancer patients. Clin Cancer Res 12, 4403s–4408s. 16857818 10.1158/1078-0432.CCR-06-0099

[23] Prim N , Legrain M , Guerin E , Mennecier B , Weingertner N , Voegeli AC , Guenot D , Maugard CM , Quoix AE and Beau‐Faller M (2014) Germ‐line exon 21 EGFR mutations, V843I and P848L, in nonsmall cell lung cancer patients. Eur Respir Rev 23, 390–392. 25176975 10.1183/09059180.00009313 PMC9487309

[24] Gimbrone NT , Sarcar B , Gordian ER , Rivera JI , Lopez C , Yoder SJ , Teer JK , Welsh EA , Chiaporri AA , Schabath MB *et al* (2017) Somatic mutations and ancestry markers in hispanic lung cancer patients. J Thorac Oncol 12, 1851–1856. 10.1016/j.jtho.2017.08.019 PMC5701827 28911955

[25] Jiang J , Greulich H , Janne PA , Sellers WR , Meyerson M and Griffin JD (2005) Epidermal growth factor‐independent transformation of Ba/F3 cells with cancer‐derived epidermal growth factor receptor mutants induces gefitinib‐sensitive cell cycle progression. Cancer Res 65, 8968–8974. 16204070 10.1158/0008-5472.CAN-05-1829

[26] Pradhan A , Lambert QT , Griner LN and Reuther GW (2010) Activation of JAK2‐V617F by components of heterodimeric cytokine receptors. J Biol Chem 285, 16651–16663. 20363735 10.1074/jbc.M109.071191 PMC2878064

[27] Kuenzi BM , Remsing Rix LL , Stewart PA , Fang B , Kinose F , Bryant AT , Boyle TA , Koomen JM , Haura EB and Rix U (2017) Polypharmacology‐based ceritinib repurposing using integrated functional proteomics. Nat Chem Biol 13, 1222–1231. 28991240 10.1038/nchembio.2489 PMC5909815

[28] Chamrad I , Rix U , Stukalov A , Gridling M , Parapatics K , Muller AC , Altiok S , Colinge J , Superti‐Furga G , Haura EB *et al* (2013) A miniaturized chemical proteomic approach for target profiling of clinical kinase inhibitors in tumor biopsies. J Proteome Res 12, 4005–4017. 23901793 10.1021/pr400309p PMC4127982

[29] Sumi NJ , Kuenzi BM , Knezevic CE , Remsing Rix LL and Rix U (2015) Chemoproteomics reveals novel protein and lipid kinase targets of clinical CDK4/6 inhibitors in lung cancer. ACS Chem Biol 10, 2680–2686. 26390342 10.1021/acschembio.5b00368 PMC4684772

[30] Sordella R , Bell DW , Haber DA and Settleman J (2004) Gefitinib‐sensitizing EGFR mutations in lung cancer activate anti‐apoptotic pathways. Science 305, 1163–1167. 15284455 10.1126/science.1101637

[31] Kobayashi S , Canepa HM , Bailey AS , Nakayama S , Yamaguchi N , Goldstein MA , Huberman MS and Costa DB (2013) Compound EGFR mutations and response to EGFR tyrosine kinase inhibitors. J Thorac Oncol 8, 45–51. 10.1097/JTO.0b013e3182781e35 PMC3531043 23242437

[32] Borras E , Jurado I , Hernan I , Gamundi MJ , Dias M , Marti I , Mane B , Arcusa A , Agundez JA , Blanca M *et al* (2011) Clinical pharmacogenomic testing of KRAS, BRAF and EGFR mutations by high resolution melting analysis and ultra‐deep pyrosequencing. BMC Cancer 11, 406. 21943394 10.1186/1471-2407-11-406 PMC3192787

[33] Han B , Zhou X , Zhang RX , Zang WF , Chen ZY , Song HD , Wan HY and Zheng CX (2011) Mutations of the epidermal growth factor receptor gene in NSCLC patients. Oncol Lett 2, 1233–1237. 22848293 10.3892/ol.2011.366 PMC3406548

[34] Faehling M , Schwenk B , Kramberg S , Eckert R , Volckmar AL , Stenzinger A and Strater J (2017) Oncogenic driver mutations, treatment, and EGFR‐TKI resistance in a Caucasian population with non‐small cell lung cancer: survival in clinical practice. Oncotarget 8, 77897–77914. 29100434 10.18632/oncotarget.20857 PMC5652823

[35] Ray P , Tan YS , Somnay V , Mehta R , Sitto M , Ahsan A , Nyati S , Naughton JP , Bridges A , Zhao L *et al* (2016) Differential protein stability of EGFR mutants determines responsiveness to tyrosine kinase inhibitors. Oncotarget 7, 68597–68613. 27612423 10.18632/oncotarget.11860 PMC5356576

[36] Lo HW (2010) Nuclear mode of the EGFR signaling network: biology, prognostic value, and therapeutic implications. Discov Med 10, 44–51. 20670598 PMC3637667

[37] Augustin A , Lamerz J , Meistermann H , Golling S , Scheiblich S , Hermann JC , Duchateau‐Nguyen G , Tzouros M , Avila DW , Langen H *et al* (2013) Quantitative chemical proteomics profiling differentiates erlotinib from gefitinib in EGFR wild‐type non‐small cell lung carcinoma cell lines. Mol Cancer Ther 12, 520–529. 23371860 10.1158/1535-7163.MCT-12-0880

[38] Levkowitz G , Waterman H , Ettenberg SA , Katz M , Tsygankov AY , Alroy I , Lavi S , Iwai K , Reiss Y , Ciechanover A *et al* (1999) Ubiquitin ligase activity and tyrosine phosphorylation underlie suppression of growth factor signaling by c‐Cbl/Sli‐1. Mol Cell 4, 1029–1040. 10635327 10.1016/s1097-2765(00)80231-2

[39] Han W , Zhang T , Yu H , Foulke JG and Tang CK (2006) Hypophosphorylation of residue Y1045 leads to defective downregulation of EGFRvIII. Cancer Biol Ther 5, 1361–1368. 16969069 10.4161/cbt.5.10.3226

[40] David M , Wong L , Flavell R , Thompson SA , Wells A , Larner AC and Johnson GR (1996) STAT activation by epidermal growth factor (EGF) and amphiregulin. Requirement for the EGF receptor kinase but not for tyrosine phosphorylation sites or JAK1. J Biol Chem 271, 9185–9188. 8621573 10.1074/jbc.271.16.9185

[41] Gao SP , Chang Q , Mao N , Daly LA , Vogel R , Chan T , Liu SH , Bournazou E , Schori E , Zhang H *et al* (2016) JAK2 inhibition sensitizes resistant EGFR‐mutant lung adenocarcinoma to tyrosine kinase inhibitors. Sci Signal 9, ra33. 27025877 10.1126/scisignal.aac8460 PMC4950506

[42] Kim EY , Cho EN , Park HS , Hong JY , Lim S , Youn JP , Hwang SY and Chang YS (2016) Compound EGFR mutation is frequently detected with co‐mutations of actionable genes and associated with poor clinical outcome in lung adenocarcinoma. Cancer Biol Ther 17, 237–245. 26785607 10.1080/15384047.2016.1139235 PMC4848002

[43] Zhang Y , Sun Y , Pan Y , Li C , Shen L , Li Y , Luo X , Ye T , Wang R , Hu H *et al* (2012) Frequency of driver mutations in lung adenocarcinoma from female never‐smokers varies with histologic subtypes and age at diagnosis. Clin Cancer Res 18, 1947–1953. 22317764 10.1158/1078-0432.CCR-11-2511 PMC3319848

[44] Lin MT , Mosier SL , Thiess M , Beierl KF , Debeljak M , Tseng LH , Chen G , Yegnasubramanian S , Ho H , Cope L *et al* (2014) Clinical validation of KRAS, BRAF, and EGFR mutation detection using next‐generation sequencing. Am J Clin Pathol 141, 856–866. 24838331 10.1309/AJCPMWGWGO34EGOD PMC4332779

[45] de Gunst MM , Gallegos‐Ruiz MI , Giaccone G and Rodriguez JA (2007) Functional analysis of cancer‐associated EGFR mutants using a cellular assay with YFP‐tagged EGFR intracellular domain. Mol Cancer 6, 56. 17877814 10.1186/1476-4598-6-56 PMC2064929

[46] Santamaria I , Menendez ST and Balbin M (2013) EGFR L858R mutation may go undetected because of P848L in cis mutation. J Clin Oncol 31, e420–e421. 23897956 10.1200/JCO.2012.47.3512

[47] Chang YL , Wu CT , Shih JY and Lee YC (2011) Comparison of p53 and epidermal growth factor receptor gene status between primary tumors and lymph node metastases in non‐small cell lung cancers. Ann Surg Oncol 18, 543–550. 20811949 10.1245/s10434-010-1295-6

[48] Zheng C , Sun YH , Ye XL , Chen HQ and Ji HB (2011) Establishment and characterization of primary lung cancer cell lines from Chinese population. Acta Pharmacol Sin 32, 385–392. 21372829 10.1038/aps.2010.214 PMC4002770

[49] Centeno I , Blay P , Santamaria I , Astudillo A , Pitiot AS , Osorio FG , Gonzalez‐Arriaga P , Iglesias F , Menendez P , Tardon A *et al* (2011) Germ‐line mutations in epidermal growth factor receptor (EGFR) are rare but may contribute to oncogenesis: a novel germ‐line mutation in EGFR detected in a patient with lung adenocarcinoma. BMC Cancer 11, 172. 21575252 10.1186/1471-2407-11-172 PMC3123322

[50] Kovacs E , Das R , Wang Q , Collier TS , Cantor A , Huang Y , Wong K , Mirza A , Barros T , Grob P *et al* (2015) Analysis of the role of the C‐terminal tail in the regulation of the epidermal growth factor receptor. Mol Cell Biol 35, 3083–3102. 26124280 10.1128/MCB.00248-15 PMC4525312

[51] Moscatello DK , Holgado‐Madruga M , Godwin AK , Ramirez G , Gunn G , Zoltick PW , Biegel JA , Hayes RL and Wong AJ (1995) Frequent expression of a mutant epidermal growth factor receptor in multiple human tumors. Cancer Res 55, 5536–5539. 7585629

[52] Lee JH , Park KS , Alberobello AT , Kallakury B , Weng MT , Wang Y and Giaccone G (2013) The Janus kinases inhibitor AZD1480 attenuates growth of small cell lung cancers *in vitro* and *in vivo* . Clin Cancer Res 19, 6777–6786. 24158701 10.1158/1078-0432.CCR-13-1110 PMC3872034

[53] Song L , Rawal B , Nemeth JA and Haura EB (2011) JAK1 activates STAT3 activity in non‐small‐cell lung cancer cells and IL‐6 neutralizing antibodies can suppress JAK1‐STAT3 signaling. Mol Cancer Ther 10, 481–494. 21216930 10.1158/1535-7163.MCT-10-0502 PMC4084653

[54] Kim SM , Kwon OJ , Hong YK , Kim JH , Solca F , Ha SJ , Soo RA , Christensen JG , Lee JH and Cho BC (2012) Activation of IL‐6R/JAK1/STAT3 signaling induces *de novo* resistance to irreversible EGFR inhibitors in non‐small cell lung cancer with T790M resistance mutation. Mol Cancer Ther 11, 2254–2264. 22891040 10.1158/1535-7163.MCT-12-0311

[55] Gao SP , Mark KG , Leslie K , Pao W , Motoi N , Gerald WL , Travis WD , Bornmann W , Veach D , Clarkson B *et al* (2007) Mutations in the EGFR kinase domain mediate STAT3 activation via IL‐6 production in human lung adenocarcinomas. J Clin Investig 117, 3846–3856. 18060032 10.1172/JCI31871 PMC2096430

[56] Sen B , Saigal B , Parikh N , Gallick G and Johnson FM (2009) Sustained Src inhibition results in signal transducer and activator of transcription 3 (STAT3) activation and cancer cell survival via altered Janus‐activated kinase‐STAT3 binding. Cancer Res 69, 1958–1965. 19223541 10.1158/0008-5472.CAN-08-2944 PMC2929826

[57] Cai L , Zhang G , Tong X , You Q , An Y , Wang Y , Guo L , Wang T , Zhu D and Zheng J (2010) Growth inhibition of human ovarian cancer cells by blocking STAT3 activation with small interfering RNA. Eur J Obstet Gynecol Reprod Biol 148, 73–80. 19880237 10.1016/j.ejogrb.2009.09.018

[58] Burke WM , Jin X , Lin HJ , Huang M , Liu R , Reynolds RK and Lin J (2001) Inhibition of constitutively active Stat3 suppresses growth of human ovarian and breast cancer cells. Oncogene 20, 7925–7934. 11753675 10.1038/sj.onc.1204990

[59] Yu HA , Tian SK , Drilon AE , Borsu L , Riely GJ , Arcila ME and Ladanyi M (2015) Acquired resistance of EGFR‐mutant lung cancer to a T790M‐specific EGFR inhibitor: emergence of a third mutation (C797S) in the EGFR tyrosine kinase domain. JAMA Oncol 1, 982–984. 26181354 10.1001/jamaoncol.2015.1066 PMC4665629

[60] Yun CH , Mengwasser KE , Toms AV , Woo MS , Greulich H , Wong KK , Meyerson M and Eck MJ (2008) The T790M mutation in EGFR kinase causes drug resistance by increasing the affinity for ATP. Proc Natl Acad Sci USA 105, 2070–2075. 18227510 10.1073/pnas.0709662105 PMC2538882

[61] Remsing Rix LL , Kuenzi BM , Luo Y , Remily‐Wood E , Kinose F , Wright G , Li J , Koomen JM , Haura EB , Lawrence HR *et al* (2014) GSK3 alpha and beta are new functionally relevant targets of tivantinib in lung cancer cells. ACS Chem Biol 9, 353–358. 24215125 10.1021/cb400660a PMC3944088

