# Supplementary material for: Characterization of epidermal growth factor receptor (EGFR) P848L, an unusual EGFR variant present in lung cancer patients, in a murine Ba/F3 model
Source: FEBS Open Bio. 2019 Sep 7;9(10):1689–704. doi: 10.1002/2211-5463.12702 (PMC6768113; doi:10.1002/2211-5463.12702)

Supplementary Figures

Characterization of epidermal growth factor receptor (*EGFR*) P848L, an unusual EGFR variant present in lung cancer patients, in a murine Ba/F3 model

Sarcar, *et. al.*

Supplementary Figure Legends

Figure S1. Sensitivity status of EGFR mutants to first and third generation TKIs. (A) Ba/F3 cells, stably expressing mutant EGFR proteins, were seeded in 12-well plates (100,000 cells/well), incubated either with vehicle control (DMSO) or with the 3, 10, 30, 90 nM of erlotinib for 3 days. Generally, cell viability was determined using a hemocytometer and the trypan blue dye-exclusion assay. (B, C, D, and E) The sensitivity of *EGFR* variants (L858R, T790M, S768insVGH, and P848L) to TKIs (erlotinib, osimertinib, and CL-387,785) was evaluated using a high-throughput Cell Titer-Glo. Briefly, 1000 cells/well were seeded into black clear-bottom 384-well plates. Inhibitors were added immediately after seeding. Cells were incubated for 3 days prior to analysis with Cell Titer-Glo Luminescent reagent. Plates were read on an M5 Spectramax plate reader; cell viability was normalized to vehicle-treated wells and fit to a sigmoidal dose-response curve using Graph Pad Prism 6. All the experiments were performed in triplicate. Errors bars represent standard deviation.

Figure S2. P848L does not physically associate with c-Cbl. (A) L858R and P848Lexpressing Ba/F3 cells were serum-starved for 24 hrs. prior to stimulation with 100 ng/mL of EGF. Control cells (-) were serum starved, but not stimulated with EGF. 1 mg of whole cell lysates were immunoprecipitated with an EGFR antibody, then Immunobloting was done with the anti-c-Cbl antibody (upper panel). The membrane was stripped and reprobed with an anti-EGFR antibody which recognizes EGFR as control (lower panel). c-Cbl interacts with L858R EGFR in Ba/F3 cells in the presence or absence of EGF. No physical association between P848L EGFR and c-Cbl was detected in EGF induced P848L Ba/F3 cells. (B), (C) Pixel densities of phospho RTK array spots for L858R and P848L respectively against pEGFR and pPDGFR were analyzed by Image J software.

Figure S3. **L858R, S768insVGH, and P848L differ in sensitivity to targeted compounds.** Cells were treated for 72 h with 0.1μM and 1μM of each compound in biological duplicate and viability was determined using CellTiterGlo. Unsupervised hierarchical clustering of the phenotypic drug screen of 300 compounds at 0.1μM (A) and 1μM (B) in L858R, S768insVGH and P848L expressing Ba/F3 cell lines. The clustering was performed independently for each drug concentration so the order of compounds in each panel is different. Drug names and target are not intended to be legible. The first principal component was calculated for both the low (0.1μM) (C) and high (1μM) (D) conc. of compounds treatment and the top 30 drugs contributing to the variance were displayed. Hierarchical clustering was performed on the drug matrix using the Ward linkage method and Euclidean distance metric. Growth inhibition was normalized by Z-score and visually represented on a scale from -3 (blue) to 3 (red) standard deviations from the mean.

Figure S4. Inhibition of STAT3 signaling upon treatment with JAK1/2 inhibitors in L858R and P848L expressing Ba/F3 cells. Ba/F3 cells expressing either L858R (A) or P848L (B) *EGFR* mutation were treated for 1 hr. with the indicated concentrations of drugs and phosphorylation at Tyr 705 of STAT3 was measured by Western blotting. Total STAT3 and actin served as loading controls for each experiment. (C) Ba/F3 cells expressing P848L *EGFR* mutation were treated with 1µM and 5µM of AZD1480 for 1hr to measure phospho-EGFR at Tyr992, p-STAT3, and p-ERK by Western blotting. Total EGFR, STAT3, ERK, and actin served as loading controls for each experiment. Images are representative of three independent experiments.

Figure S5. Kinase activity and downstream signaling of L858R and P848L upon treatment with erlotinib for 1 hr. Ba/F3 cells expressing either L858R (A) or P848L (B) *EGFR* mutation were treated for 1 hr. with the indicated concentrations of erlotinib and phospho-EGFR at Tyr992, phosphorylation at Tyr 705 of STAT3 and Tyr 694 at STAT5 and p-ERK was measured by Western blotting. Total EGFR, STAT3, STAT5, ERK, and actin served as loading controls for each experiment. Images are representative of three independent experiments.

Supplementary Fig. S1

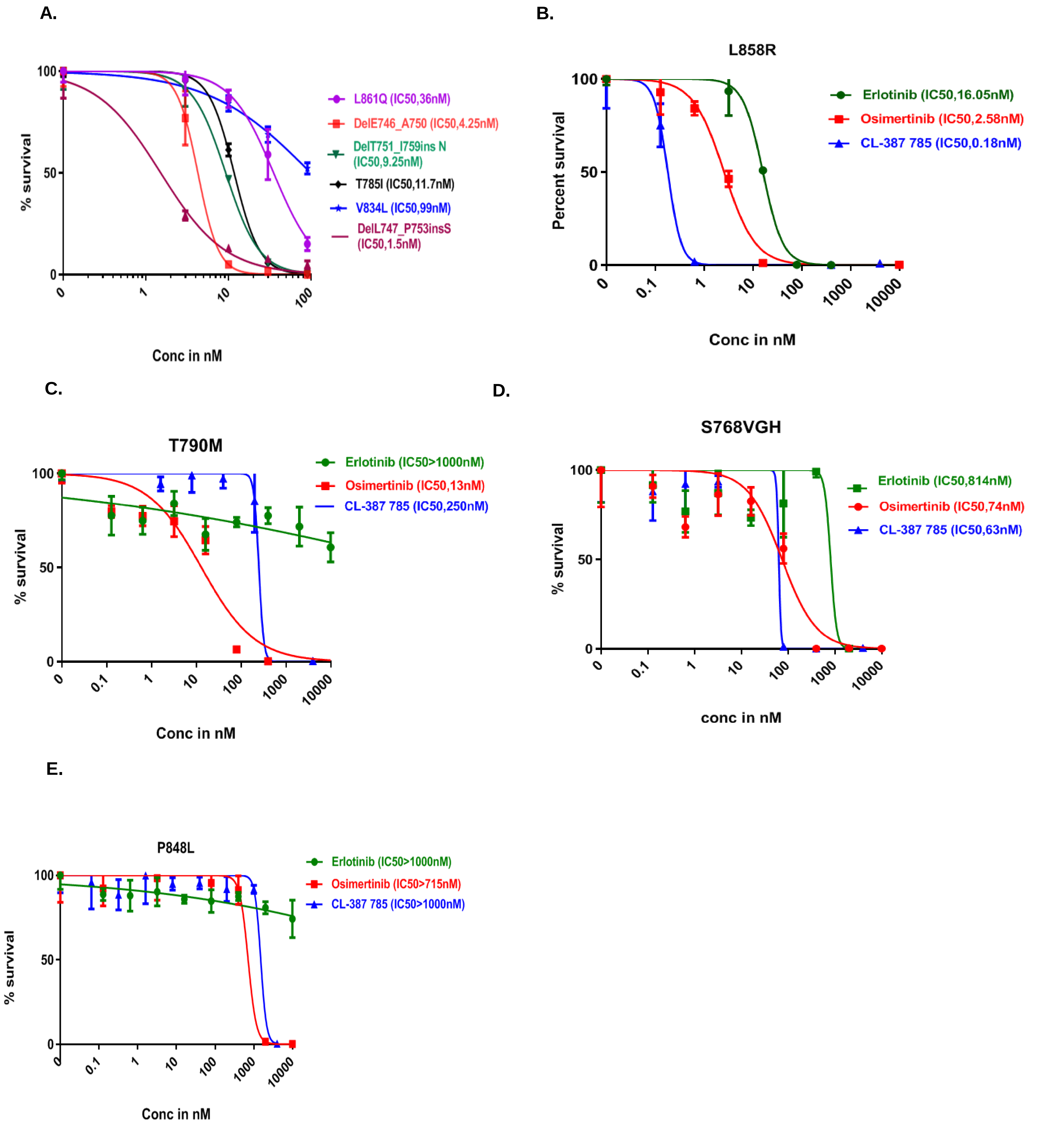

Supplementary Fig. S2

A.

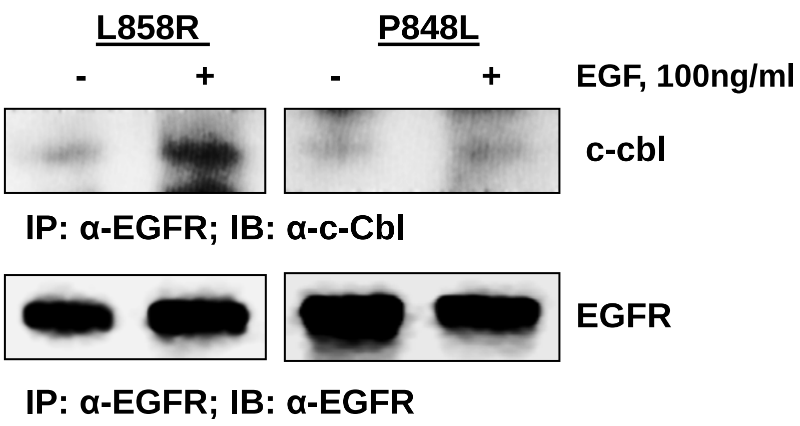

B.

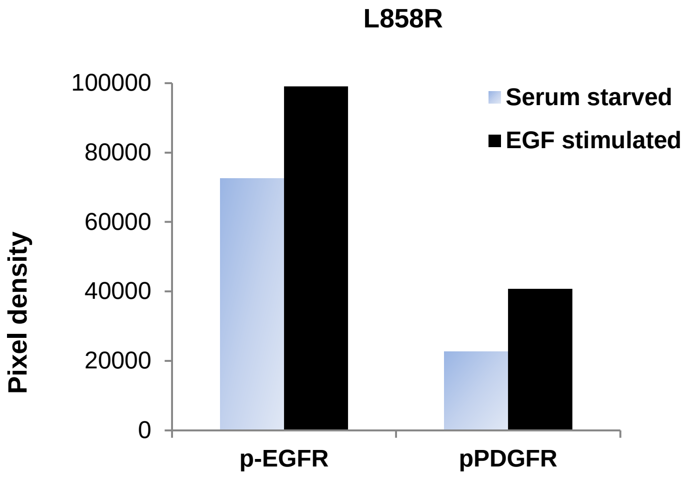

C.

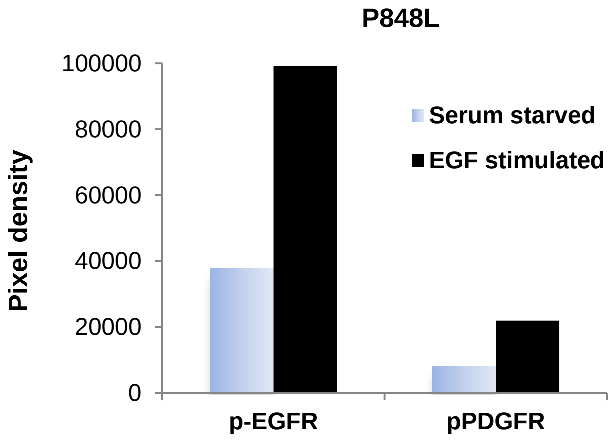

Supplementary Fig. S3

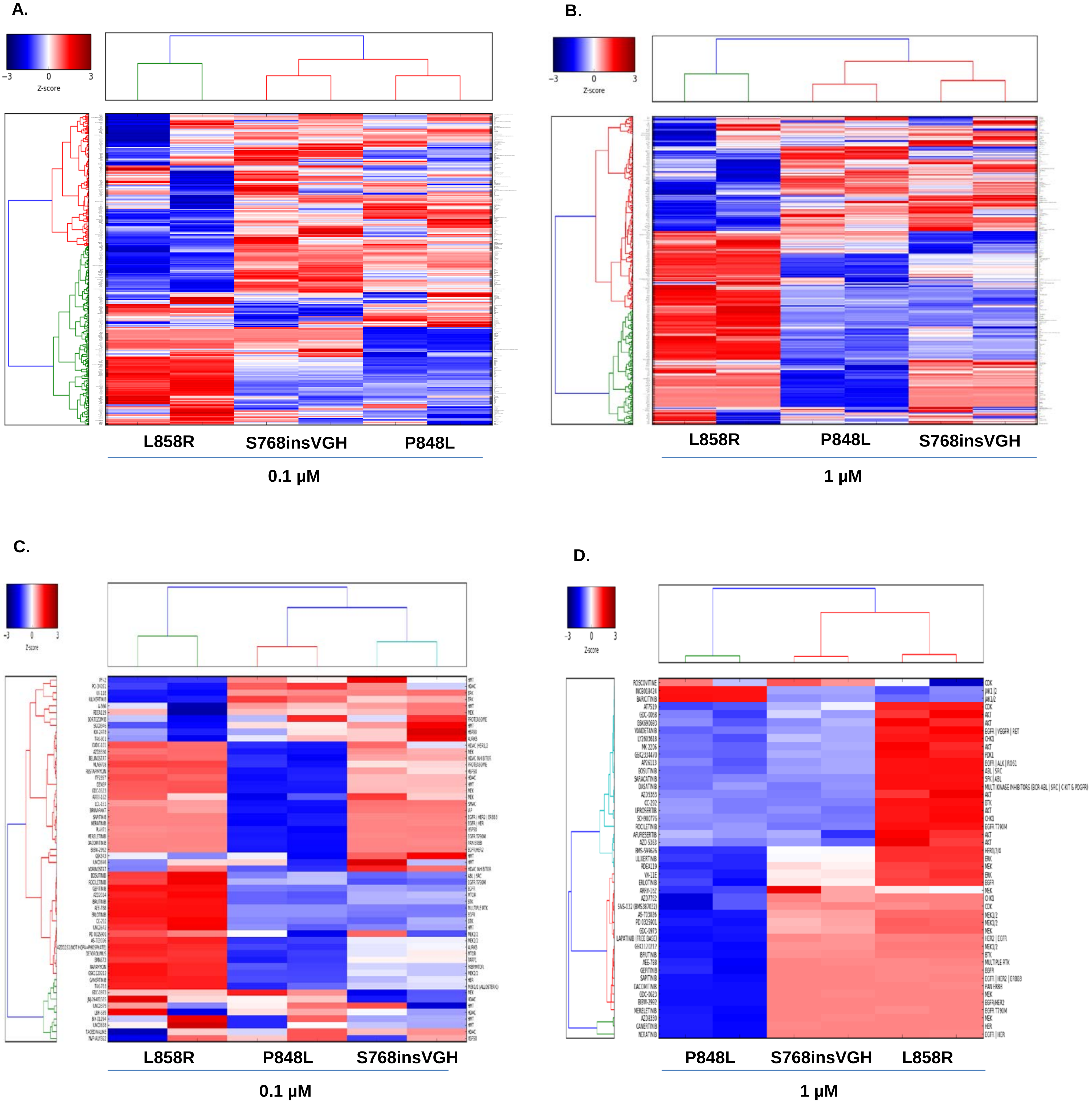

Supplementary Fig. S4

A.

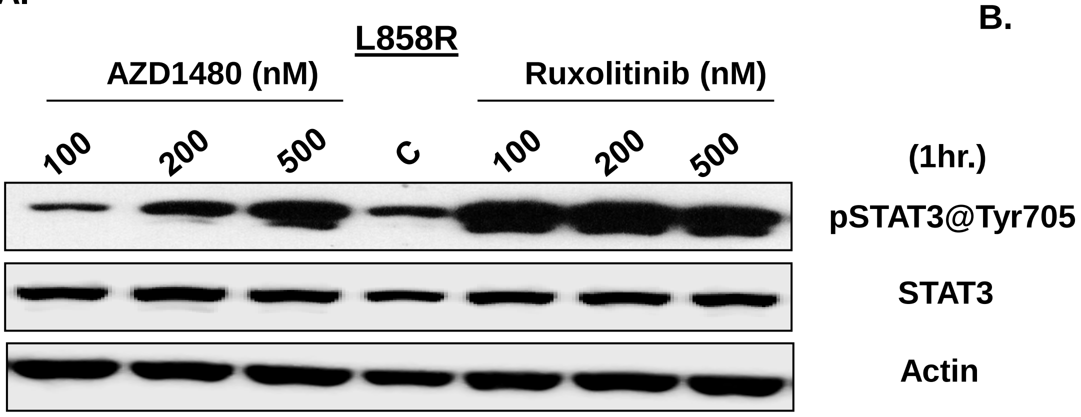

B.

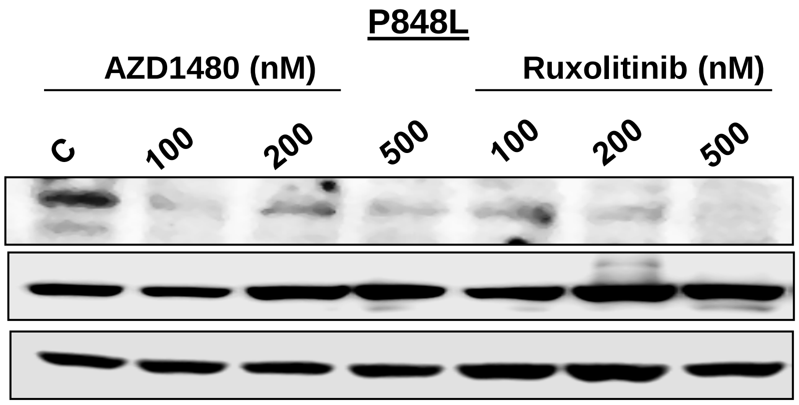

C.

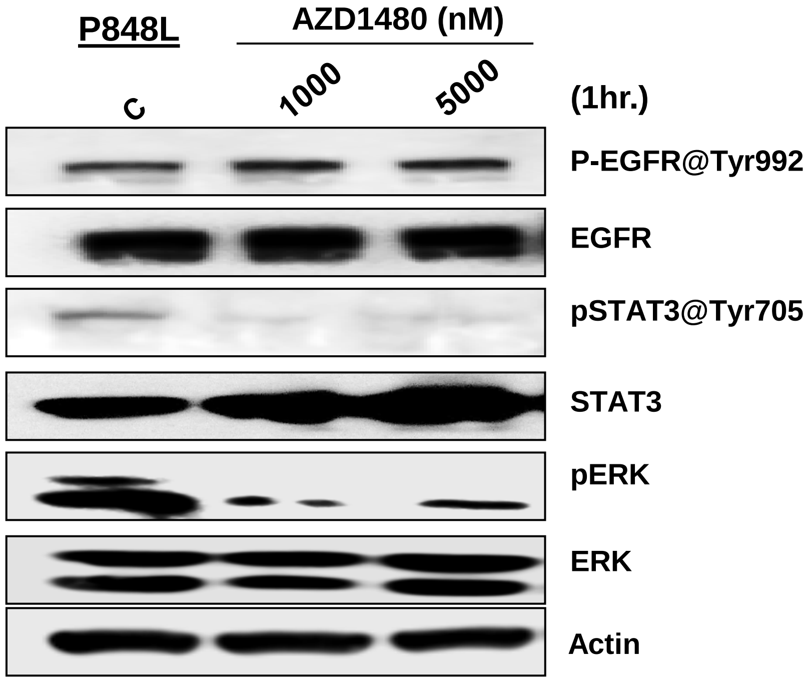

Supplementary Fig. S5

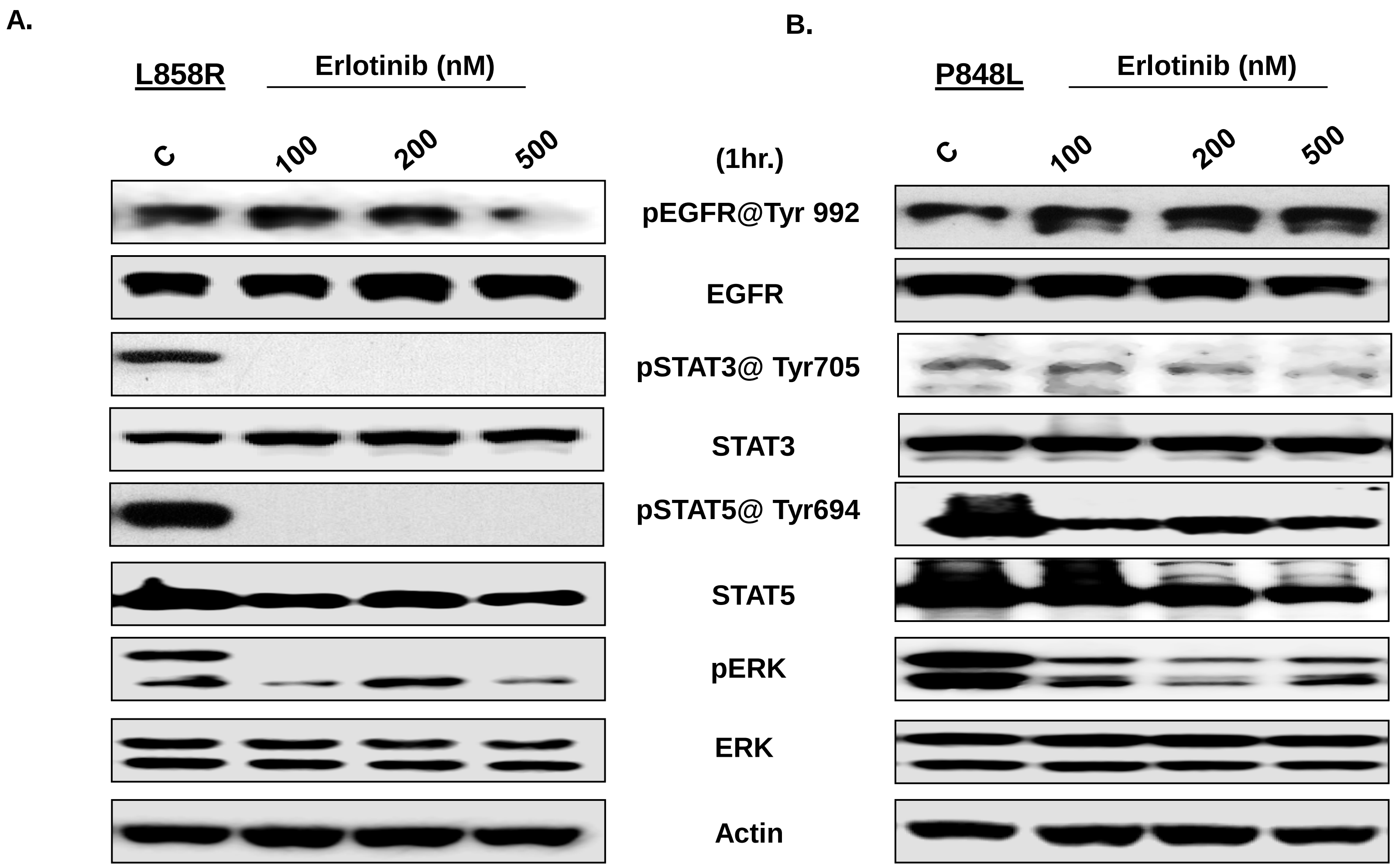

Supplement: Supplementary file 1 — Fig. S1. Sensitivity status of EGFR mutants to first and third generation TKIs. Fig. S2. P848L does not physically associate with c‐Cbl. Fig. S3. L858R, S768insVGH, and P848L differ in sensitivity to targeted compounds. Fig. S4. Inhibition of STAT3 signaling upon treatment with JAK1/2 inhibitors in L858R and P848L expressing Ba/F3 cells. Fig. S5. Kinase activity and downstream signaling of L858R and P848L upon treatment with erlotinib for 1 h. [file FEB4-9-1689-s001.pdf]
